# Guided Autotransplantation of Impacted Canines Using a CAD/CAM Surgical Template

**DOI:** 10.3390/children10040708

**Published:** 2023-04-11

**Authors:** Soyoung Park, Haena Lee, Eungyung Lee, Taesung Jeong, Hyeonjong Lee, Jonghyun Shin

**Affiliations:** 1 Department of Pediatric Dentistry, Dental Research Institute, Pusan National University Dental Hospital, Yangsan 50612, Republic of Korea; soyoungpark85@gmail.com (S.P.); leeungyung@gmail.com (E.L.); tsjeong@pusan.ac.kr (T.J.); 2 Department of Pediatric Dentistry, Dental and Life Science Institute, School of Dentistry, Pusan National University, Yangsan 50612, Republic of Korea; haenhaen12@gmail.com; 3 Department of Prosthodontics, College of Dentistry, Yonsei University, Seoul 03722, Republic of Korea

**Keywords:** transplantation, autologous, cuspid, tooth, impacted, computer-aided design, printing, three-dimensional, osteotomy

## Abstract

Autotransplantation is a potential treatment alternative when orthodontic traction of an impacted tooth is difficult. In this article, we describe two cases of guided autotransplantation of an impacted canine using a computer-aided designed and manufactured surgical template. The impacted canine was segmented on preoperative cone-beam computed tomography images to ensure a sufficient periodontal ligament space and placement of the donor tooth with the least pressure on it. The canine was virtually transposed using a simulation program considering the adjacent teeth. The surgical template, which was connected to the occlusal stop on adjacent teeth, was designed and 3D-printed with polymer resin. The recipient site was prepared using the surgical template, followed by immediate transplantation of the surgically extracted canine into the socket. The transplanted donor tooth was positioned in planned infra-occlusion to prevent occlusal interference. It was then splinted with the adjacent teeth for initial stabilization. During follow-up, one transplanted tooth showed pulp canal obliteration and the other had suspected pulp necrosis; endodontic treatment was performed. One year after the procedure, the periradicular condition of both teeth was favorable.

## 1. Introduction

Impacted canines are often detected in pediatric dentistry. In cases of canines with a palatal ectopic tendency overlapping the lateral incisor, extraction of the primary canine before 11 years of age has been reported to improve the eruption pathway in 64–91% cases [1]. Generally, following space creation, the eruption of an impacted tooth is guided through surgical exposure and orthodontic traction [2]. However, if the impacted tooth has been displaced far from the common eruption path or blocked by a nearby anatomic structure, orthodontic traction might take longer or fail. In this case, extraction of the impacted canine is a potential treatment option. Nevertheless, canines are functionally important teeth in the dental arch. Autotransplantation can be attempted in aged patients with an open root apex, and the prognosis has been reported to be favorable [3].

Recent advancements in digital dentistry, such as cone-beam computed tomography (CBCT) and computer-aided design and manufacturing (CAD/CAM), have made enabled preoperative evaluation of impacted teeth. For example, the width and length of the donor tooth can be predicted from digital images, and a computer-aided rapid prototyping tooth model can be used to shape the recipient site instead of relying on the impacted tooth itself [4,5]. The fact that the donor tooth can be extracted after socket contouring is notable because it allows the vitality of the periodontal ligament (PDL) cells to be preserved by reducing the extraoral dry time. However, simple copying of the impacted tooth has limitations because it is difficult to determine its accurate direction and depth when positioning.

In implant prosthodontics, a surgical guide is commonly used to achieve accurate depth, direction, and angulation during abutment placement, and CAD/CAM technology is introduced for its fabrication [6]. A surgical guide has also been considered for autotransplantation. Unlike implant prosthetics, in tooth transplantation, it is important to create a recipient site wide enough for a 1–2 mm thick blood clot to cover the entire root surface of the transplanted tooth [3,7].

This report describes the design and fabrication of surgical templates produced to prevent pressure-induced damage to the PDL and promote revascularization of the pulpal space. The aim of this report was to present cases of autotransplantation using virtually planned 3D-printed surgical templates for guided osteotomy preparation of recipient sites. This method can ensure an atraumatic and precise surgical approach for autotransplantation of impacted canines.

## 2. Fabrication of Surgical Template

**Figure 1:**
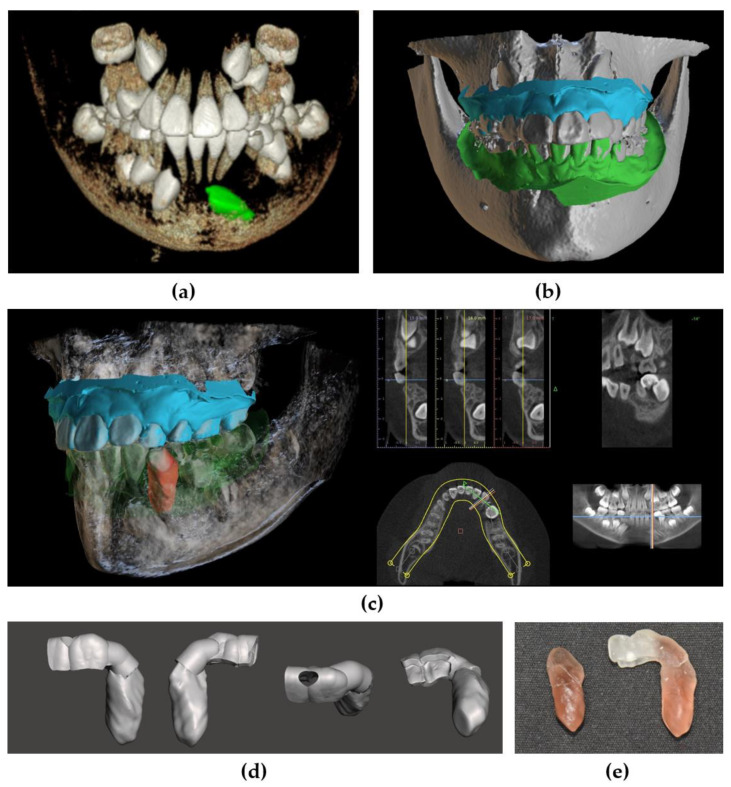
Fabrication procedure of surgical template. (**a**) Segmentation of the impacted canine; (**b**) merging of the cone-beam computed tomography and intraoral scan images; (**c**) virtual transplantation of the impacted canine to the ideal intra-arch position; (**d**) design of surgical template; and (**e**) 3D-printed canine replica and surgical template.

A preoperative 3D radiographic examination (Promax^®^, Planmeca, Helsinki, Finland) was performed using the following scanning parameters: voxel size 0.3 mm, 110 kV, 11.0 mA, 3.272 s. The CBCT DICOM images were imported into an open-source imaging software (3D slicer, https://www.slicer.org/ (accessed on 5 January 2021) [8]. Using the segmentation mode, the donor tooth or impacted canine, including the PDL space, was selected (Figure 1a). The segmented part was exported to a standard tesselation language (STL) file. STL files of scanned dental models and intraoral scans (MEDIT i500; MEDIT Corp., Seoul, Korea) were merged with CBCT images using virtual surgery planning software (Blue Sky Plan^®^, Blue Sky Bio, Libertyville, IL, USA) (Figure 1b). The ideal position, angulation, and rotation of the canine were predefined with the aid of the STL flies of the donor tooth (Figure 1c). Surgical templates for guided osteotomy were designed, including canine replicas connected to occlusal stops on adjacent teeth (Meshmixer, Autodesk, Inc., San Rafael, CA, USA) (Figure 1d). The surgical template and tooth replica were exported as STL files and sent to a 3D printer (NextDent 5100, 3D Systems, Rock Hill, SC, USA) for fabrication (Figure 1e).

## 3. Case Presentation

### 3.1. Case 1

**Figure 2:**
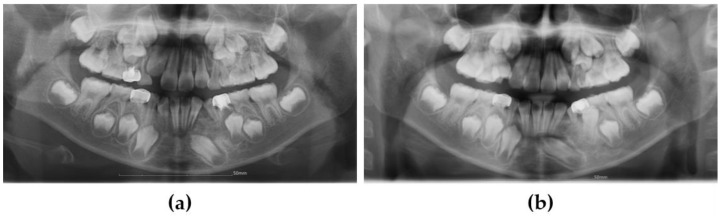
Panoramic radiographs. (**a**) At the time of the first visit, the left mandibular canine was directed to incisors, and an abnormal trabecular bone pattern was present between the lateral incisor, primary canine, and permanent canine; (**b**) Ten months after excision of the cemento-ossifying fibroma, COF, the left mandibular canine appeared impacted, and the first premolar had erupted distobuccally.

A 9-year-old boy visited our clinic with bony swelling in the left mandible. On a panoramic radiograph, a radio-mixed lesion was visible between the roots of the left mandibular lateral incisor and primary canine. The distance between the apices of the roots of the lateral incisor (#32) and primary canine (#73) had increased, and the tooth buds of the permanent canine (#33) and first premolar (#34) were displaced due to the lesion (Figure 2a). A benign neoplasm, finally diagnosed as a cemento-ossifying fibroma (COF), was excised under general anesthesia. The displaced canine and premolars were followed up, expecting spontaneous eruption. Ten months later, the first premolar emerged distobuccally; however, the canine was displaced more horizontally toward the apex of the incisors (Figure 2b). An additional CBCT image showed an abnormal trabecular bone pattern, raising concerns regarding recurrence. Furthermore, the impacted canine was positioned at the center of the alveolar bone near the mandibular symphysis. The success of orthodontic traction was uncertain, and even if attempted, it would have required a significant amount of time and cooperation from the patient and guardians. Both the patient and his parents worried about discomfort during prolonged treatment. Moreover, since the bone that would provide a path for traction was unhealthy and no additional space was needed, autotransplantation of the impacted tooth and curettage of the recurrent lesion were performed.

**Figure 3:**
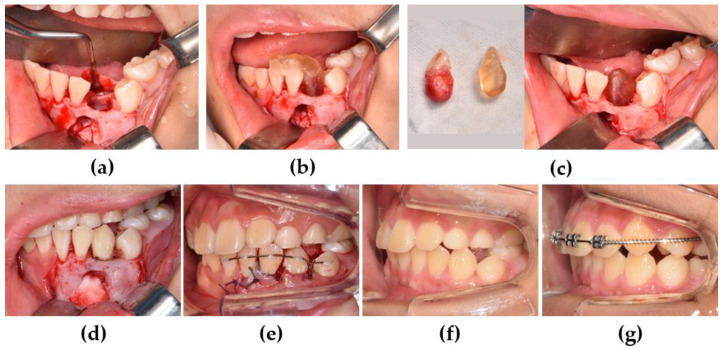
Autotransplantation of impacted left mandibular canine (Case 1): Surgical procedure (**a**–**e**). The shape and size of the 3D-printed replica is similar to those of the real extracted canine. The clinical condition at the 3-month (**f**) and 18-month follow-up (**g**) is shown.

During the surgical procedure, the primary canine was removed and a full-thickness gingival flap was reflected. The impacted canine was located immediately below the recipient site, so the upper alveolar socket was first enlarged to make room for the transplanted tooth (Figure 3a). The donor tooth was carefully extracted to avoid damage to the PDL cells and lightly wrapped with saline-soaked gauze to prevent drying. A surgical template with segmented teeth and a 3D-printed tooth replica were inserted to verify the final preparation of the recipient site (Figure 3b,c). The extracted canine was transplanted into the planned infra-occlusal position to prevent occlusion interference (Figure 3d). The gingival flap was repositioned and sutured, and nonrigid fixation of the transplanted tooth with the adjacent teeth was performed on the buccal surface for postoperative stabilization (Figure 3e). Oral antibiotics and a chlorhexidine mouth rinse were prescribed for one week. The healing process was uneventful, and the sutures were removed 10 days after surgery. At the 3-month and 18-month follow-ups, the patient showed no symptoms and had no postoperative complications (Figure 3f,g).

**Figure 4:**
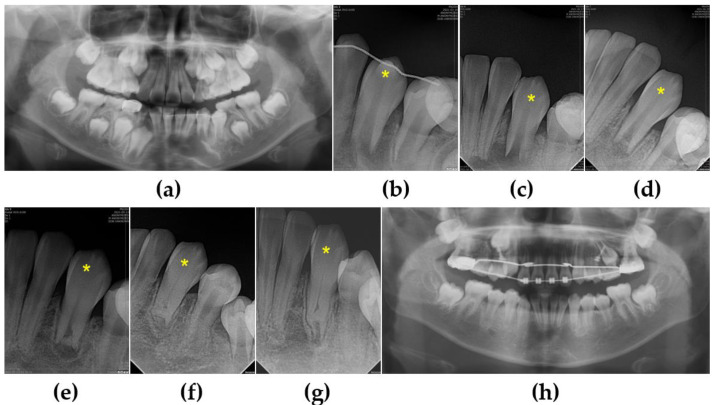
Radiographic findings. The transplanted tooth is marked with a yellow asterisk (*). Radiographs were taken on the day of autotransplantation (**a**,**b**), 1 month (**c**), 3 months (**d**), 6 months (**e**), 12 months (**f**), and 18 months (**g**,**h**) postoperatively.

The transplantation of the canine at the planned position was verified on panoramic and periapical radiographs on the day of the surgery (Figure 4a,b). Periapical radiographs were taken at the 1-, 3-, 6-, and 12-month follow-up evaluations after autotransplantation. Root canal obliteration, physiological PDL space, and lamina dura were observed (Figure 4c–f). At the 18-month follow-up, further root development was observed with no other symptom, sign of pathology, or root resorption (Figure 4g,h).

### 3.2. Case 2

**Figure 5:**
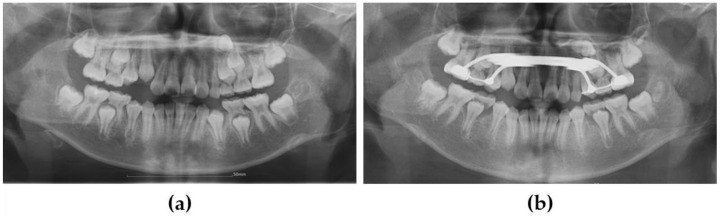
Panoramic radiographs. (**a**) The left maxillary canine (#23) has been displaced apically and horizontally and is facing forward; (**b**) rapid maxillary expansion was performed to create space for the canine.

A 10-year-old girl was referred from a local dental clinic for prolonged retention of the left maxillary primary canine and eruption disturbance at the left maxillary canine (#23). A panoramic radiograph revealed that the maxillary canine was impacted horizontally toward the anterior region and between the apex of the left maxillary premolars and nasal cavity (Figure 5a). Because the canine was impacted at the base of the alveolar bone, with the premolars blocking its eruption path, orthodontic traction was challenging and potentially ineffective as a treatment option. Therefore, we decided to perform autotransplantation of the canine with comprehensive orthodontic treatment. During preoperative orthodontic treatment, a rapid maxillary expansion appliance and a fixed orthodontic appliance were used for space gain and alignment (Figure 5b).

**Figure 6:**
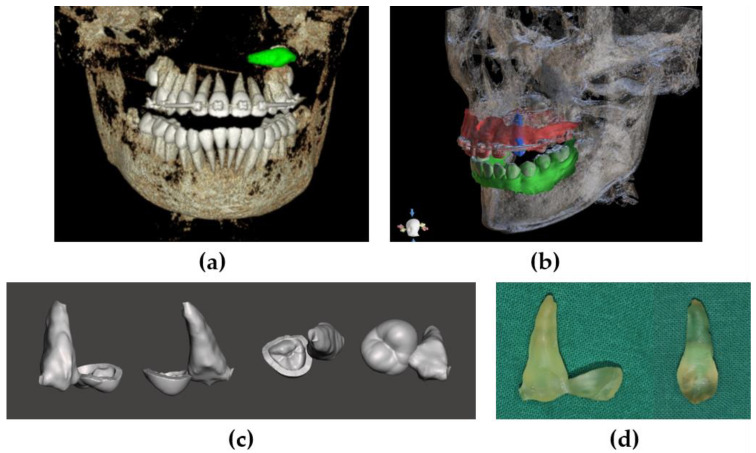
Surgery planning and fabrication of a surgical template. (**a**) segmentation of impacted canine; (**b**) virtual surgery planning using CBCT and intraoral scan images; (**c**) design of a surgical template; (**d**) 3D-printed surgical template and tooth replica.

To fabricate a surgical template, the impacted canine was segmented from the CBCT image, and transplantation was simulated using surgery planning software. In this case, the roots of the lateral incisor and first premolar were so close to the planned recipient site that they had to be repeatedly evaluated on sagittal, coronal, and axial CBCT views (Figure 6a,b). The radicular portion of the canine replica was designed to be larger than the real canine root by including a wide PDL space to allow for passive positioning of the donor tooth without any compression around the root when positioned. Occlusal stops on the adjacent teeth were connected to the tooth replica (Figure 6c). The canine replica and surgical template were 3D printed using a polymer resin (Figure 6d).

**Figure 7:**
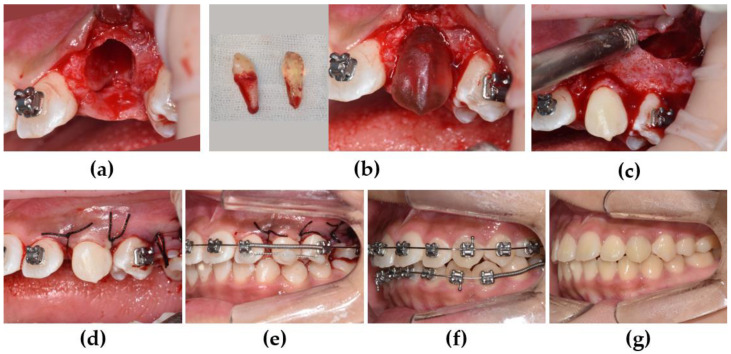
Autotransplantation of impacted left maxillary canine (Case 2): Surgical procedure (**a**–**e**). The shape and size of the 3D-printed replica is similar to those of the real extracted canine. The clinical condition at the 6-month (**f**) and 1-year follow-up (**g**) is shown.

After extracting the primary canine and raising a full-thickness flap, the alveolar socket was shaped using drills for implant prostheses. The direction and depth were determined using a surgical template (Figure 7a,b). The impacted donor tooth was atraumatically extracted to prevent damage to the PDL cells and immediately transplanted into the prepared recipient site (Figure 7c). Infraocclusion of the transplanted canine was set to prevent occlusal interference. The transplanted and adjacent teeth were non-rigidly splinted following suturing of the gingival flap (Figure 7d,e). Oral antibiotics and chlorhexidine mouth rinse were prescribed. The healing process was uneventful, and the sutures were removed seven days postoperatively. At three months after autotransplantation, pulp vitality loss was suspected based on radiographs. Root canal treatment was then performed. Following periodontal healing, an orthodontic bracket was bonded to canine. The patient showed no other clinical or radiographic symptoms during postoperative orthodontic treatment, which was completed 1 year after canine autotransplantation (Figure 7f,g).

**Figure 8:**
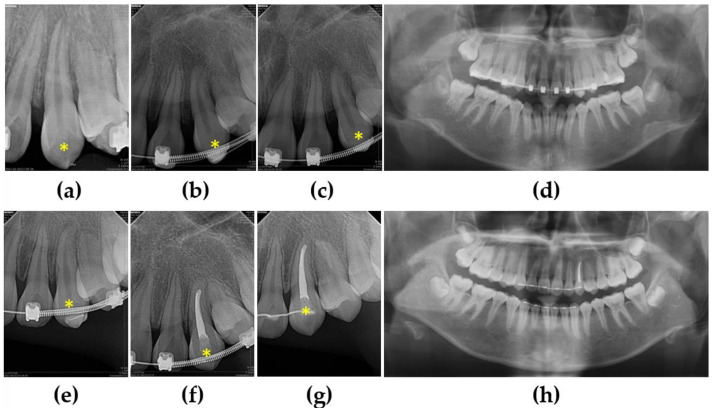
Radiographic examinations. The transplanted tooth is indicated with a yellow asterisk (*). Radiographs obtained on the day of autotransplantation (**a**), 1 week (**b**), 1 month (**c**), 3 months (**d**,**e**), 5 months (**f**), 14 months (**g**), and 2 years (**h**) postoperatively.

A periapical radiograph taken intraoperatively showed that the donor tooth (indicated with a yellow asterisk) was positioned as planned (Figure 8a). At the 1-week and 1-month follow-ups, periapical pathology was suspected but the patient had no symptoms (Figure 8b,c). Two months after, however, there was periapical radiolucency on the panorama and intraoral standard radiographs (Figure 8d,e). Periapical healing and normal lamina dura were observed 1 month after root canal treatment (Figure 8f). Two months after completion of orthodontic treatment and 14 months after autotransplantation, the periapical radiograph showed no other sign of pathology, such as root resorption (Figure 8g), and maintained a normal condition at the 2-year follow-up (Figure 8h).

## 4. Discussion

Both periodontal and pulpal recovery are important factors for good prognosis, and close examination of clinical and radiographic features is needed at every follow-up [9,10]. An immature permanent tooth has been reported to have a better prognosis when replanted owing to its “young and strong” pulp. A wide apical foramen allows immediate and spontaneous revascularization of the radicular pulp and helps maintain tooth vitality [3,11]. In Case 1, using the Nolla method [12], the root development of the donor tooth was estimated between stages 7 and 8. Pulp canal obliteration and further root development, indicating pulp vitality, appeared during the follow-up, not related to the COF excision history. However, in Case 2, pulp necrosis was suspected 3 months after autotransplantation. Because the root development of the donor was almost complete despite the opening at the apex, it was inferred that the narrow apical foramen allowed limited blood inflow. In this case, close examination of the pulp and periapical condition enabled timely endodontic treatment; therefore, the prognosis was acceptable on preventing infection. Based on the conservative concept, intervention to the pulp might be better when pulp necrosis and periapical infection are evident than when preventive endodontic treatment is performed for teeth with a closed apex. Tsukiboshi [3] recommended that pulp regeneration should be anticipated if Hertwig’s root sheath is present. Considering the possibility of pulp revascularization, autotransplantation might be recommended for teeth before Nolla developmental stage 9.

Maintenance of PDL cell vitality and prevention of infection are other important factors for tooth transplantation. According to the guidelines of the International Association of Dental Trauma in 2020, replantation of avulsed teeth in 15 min can help preserve most viable PDL cells [13]. An advantage of the surgical template is that it minimizes the extraoral dry time of the donor tooth by helping to prepare the recipient site before the extraction of the impacted tooth. Moreover, aseptic conditions of the operation field and oral antibiotics after surgery decrease risk of infection; complications, such as inflammatory external root resorption, did not occur in either case. Oral hygiene care during the early gingival healing stage is also important. Therefore, postoperative instructions for patients and guardians should be mandatory.

In the present two cases, when designing the surgical template, we focused on the shape of the tooth replica, which had a larger root than that of the real canine. During segmentation, the area was cropped to include both the canine and its PDL space. A larger tooth replica was used for osteotomy of the recipient site, which enabled a blood clot to fill around the transplanted donor tooth to allow spontaneous revascularization of the pulp. Passive positioning with low pressure in a spacious alveolar socket is considered effective for initial PDL reattachment [3]. However, limitless osteotomy is not recommended due to risks of damage to the teeth adjacent to the recipient site and poor initial stabilization. A surgical template designed for the tooth replica and connected to the adjacent teeth with occlusal stops can guide the mesiodistal and buccolingual angulation and depth of the socket during osteotomy and thereby protect the adjacent teeth.

The use of a surgical template for guided osteotomy, which was based on the concept of surgical guides for implant placement, has been reported for autotransplantation of the mandibular secondary premolar to replace a maxillary incisor lost due to dental trauma. A series of templates for implant drills with different diameters have been used to guide the path and depth of recipient site preparation. Surgical templates enable precise osteotomy, as they are almost identical to the planned shape [14]. When compared to the use of a “template series”, the surgical template used in our cases had some limitations in the precise realization of the surgery plan. First, osteotomy performance was dependent on the resistant tactile sense when the template was inserted in the alveolar socket, so the prepared recipient site was thought to be a little wider than planned. Second, this case report only showed short-term follow up under 2 years. Additional evaluations on long term prognosis, effectiveness, and efficiency would be needed. Third, the surgical procedures needed to be performed under general anesthesia in both cases in accordance with the patients’ cooperation.

In this study, the transplanted tooth was splinted for initial stabilization using a nickel-titanium wire and resin bonding. Flexible fixation is recommended to avoid replacement root resorption and the recovery of transplanted PDL cells. In cases of tooth crowding, preoperative orthodontic treatment was required for space gain. The orthodontic bracket slot had clearance when thin wire was inserted, allowing a light force to the transplanted tooth during mastication and from the tongue [15]. In Case 2, bonding the bracket to the transplanted canine and placement of an archwire might have been a more comfortable and convenient option.

We reported two cases of guided autotransplantation using surgical templates fabricated based on donor tooth replicas and occlusal stops on adjacent teeth using virtual surgery planning. Despite some limitations mentioned above, the simple design and fabrication process of the template are thought quite intuitive and clinically useful. Upgrading to a simple but more planned shape of template such as a “template series”, guiding the osteotomy drill for a more convenient surgical process, could be attempted in future cases. Keeping within the permissible irradiation range, the 3D radiographs taken right after surgery and during follow-up might help in evaluating the operation itself and prognosing its success. These radiographic records could be useful data for a retrospective survey of pre- and post-operative factors, such as a quantitative analysis of the change in alveolar bone at the recipient site and an evaluation of surgery accuracy.

In cases of superficial impaction, the laser is a viable surgical alternative because of its many intraoperative and postoperative patient comfort advantages, due to its minimal intervention [16]. However, treatment of a deeply impacted cuspid is challenging due to the limited options for a treatment plan, such as: orthodontic traction with surgical exposure, tooth transplantation, extraction of permanent tooth, or no intervention [17]. Initial canine position is the most important consideration and factors, such as age of patient, cooperative ability, predicted treatment period, and cost, also need to be included in decision-making. If tooth transplantation has failed, prosthesis such as an implant or crown-and-bridge might be needed when edentulous space has to be maintained until adulthood. Successful autotransplantation of impacted teeth might require less treatment time and help improve the quality of life of patients and their parents. Digital dentistry, including CAD/CAM, may promote efficient treatment of impacted teeth.

## 5. Conclusions

Surgical templates fabricated using CAD/CAM were successfully applied for the autotransplantation of impacted canines with minimal complications. We expected that the advances in the template design would make the surgical procedure easier and more precise. A favorable prognosis of the transplanted tooth may be ensured through suitable case selection, intraoperative infection control, and careful postoperative evaluations.
